# Immunoglobulin A Nephropathy With Associated Thrombotic Microangiopathy: Biopsy and Clinical Case Series

**DOI:** 10.1177/11795476261427772

**Published:** 2026-03-03

**Authors:** Ryan Fekrat, Arif Nihat Demirci, Caroline Gee, Sohrab Kharabaf, Mina Tadros, Matthew Nguyen, Dao Le, Omid Vadpey, Jonathan Zuckerman, Ramy Hanna

**Affiliations:** 1 Department of Medicine, Division of Nephrology, Hypertension, and Renal Transplant, University of California, Irvine, USA; 2 Department of Medicine, University of California, Irvine, USA; 3 David Geffen School of Medicine, Department of Pathology and Lab Medicine, University of California, Los Angeles, USA

**Keywords:** thrombotic microangiopathy (TMA), complement dysregulation, immunoglobulin A (IGA) nephropathy, case series, case report

## Abstract

**Introduction:**

IgA nephropathy (IgAN) with concurrent thrombotic microangiopathy (TMA) is an unusual combination of pathological findings that is associated with severe hypertension, proteinuria, and lower estimated glomerular filtration rate (eGFR). This series presents 7 patients with kidney biopsies demonstrating IgAN and concomitant TMA and presents a link by way of complement disorder.

**Case presentation:**

Seven patients all presented as IgAN with associated TMA coinciding with hypertension, proteinuria, and low eGFR. Six patients showed tubular atrophy or interstitial fibrosis and 5 of those 6 showed >50% of renal cortex containing tubular atrophy or interstitial fibrosis. Variations of low serum C3, high serum C4, elevated IgA or IgM proteins, and elevated CH50 were found in 6 of the 7 patients.

**Conclusions:**

Chronic changes seen like interstitial fibrosis and tubular atrophy appear to show a subacute or chronic nature of IgAN with TMA. The low serum C3, high C4, elevated CH50, suggests complement activation in endothelial and mesangial from IgG-IgA complexes known to occur in IgAN. This phenotype of IgAN with TMA may represent a more unique IgAN phenotype with a more severe clinical course and be indicative of underlying complement dysregulation.

## Introduction

Thrombotic Microangiopathies (TMAs) are clinical syndromes characterized by disordered coagulation and complement regulation, often resulting in microangiopathic hemolysis, microvascular thrombosis and multisystem organ failure.^1^ TMAs are difficult to diagnose given that there are no specific bioassays, and some varieties are organ system limited (ie, renal limited). Therefore, diagnosing TMA can prove especially challenging. Kidney biopsy may be helpful to diagnose systemic TMA but is crucial to determine the diagnosis in renal limited TMA.^1^

Kidney biopsies in TMA are characterized by thrombosis and normally show 2 broad forms of histopathological findings, the first form being arteriolar and arterial involvement and the second form being glomerular involvement.^2^ Low haptoglobin, elevated lactate dehydrogenase (LDH), schistocytes on peripheral smear and increased reticulocytes are among the commonly recognized serological laboratory findings in systemic TMA.^1^ A systemic TMA has a broad differential diagnosis^1^: including ADAMTS13 Deficiency–Associated TMA (thrombotic thrombocytopenic purpura-TTP), Shiga Toxin–Mediated TMA (STEC-HUS), Drug-Induced TMA, Complement-Mediated TMA (CM-TMA, also known as atypical hemolytic syndrome-aHUS), and secondary idiopathic TMA.^1^ IgA Nephropathy rarely triggers TMA, but it has been reported.^1^

Immunoglobulin A nephropathy (IgAN) is the most common glomerulonephritis worldwide^3^ and can only be definitely diagnosed on kidney biopsy demonstrating IgA dominant mesangial immune complex deposits.^4^ Complement activation and dysregulation, predominantly of lectin and alternative complement pathways, has emerged as an important component of the emerging multi-hit IgAN pathogenesis model.

IgAN with concurrent TMA has been reported in up to 53% of IgAN cases of cohorts with greater than 100 patients.^2^ Prompt recognition and treatment of concomitant TMA in IgAN presents untapped utility. In this report, we present a series of 7 patients, serology and histopathology, to highlight a complement mediated link between IgAN and TMA.

## Case Presentation

Seven patients with concurrent IgA nephropathy and histologic features of TMA were identified. The average as of the cohort was 41.6 ± 8.1 years with 5/7 being male and 4/7 being Hispanic (Table 1). One of the patients showed evidence of systemic TMA by histology; while the other 6 patients had renal limited TMA. Average initial serum creatinine for all patients was 5.17 ± 4.74 mg/dl, average initial urine protein on 24 hours urine test was 6.75 ± 5.11 g/day, and 5/7 of patients had evidence of hematuria (Table 2).

**Table 1.** Demographic Summary.

Patient #	Age	Gender	Reported ethnicity	Comorbid condition	Outcome	Medications
1	37	Male	Hispanic	ESRD secondary to biopsy-proven IgA nephropathy, HTN, HLD	Transplant	Carvedilol, hydralazine, nifedipine
2	37	Female	Asian	ESRD with IgA nephropathy	Transplant	Carvedilol, prednisone, tacrolimus
3	29	Male	Hispanic	HTN	BP uncontrolled on pharmacotherapy	Carvedilol, hydralazine, nifedipine, prednisone
4	47	Female	Asian	HTN of ESRD	On dialysis	Furosemide, hydralazine, lisinopril, metoprolol (switched to carvedilol)
5	42	Male	Hispanic	HTN	BP controlled on pharmacotherapy	Hydralazine, carvedilol, nifedipine
6	54	Male	Hispanic	HTN, DM	Recovered and maintained on pharmacological (Bumex) and supportive therapy	Nifedipine, Metformin, Aspirin, Bumex
7	45	Male	Asian	Vitamin D deficiency; Dyslipidemia	Multiple trials of ace inhibitor (on and off due to dry cough), one episode of IgA nephropathy flare suppressed with ACEi and Plaquenil	ACEi (Benazepril) Farxiga

Abbreviations: BP, blood pressure; DM, diabetes mellitus; ESRD, end-stage renal disease; HLD, hyperlipidemia; HTN, hypertension.

**Table 2.** Hematological Labs and Clinical Findings.

Patient #	Initial eGFR ml/min per 1.73 m^2^	Post-Treatment eGFR ml/min per 1.73 m^2^	Doubled SCr?	Initial urine protein g/day	Post-treatment urine protein g/day	Hematuria?	Hemoglobin g/dL	LDH (U/L)	Haptoglobin (mg/dL)	Platelets (PLTe9/L)
1	12	5	Y	13.1	-	Yes	12	176	246	215
2	3	Above 60	N	3.48	0.2	Yes	8.1	-	-	280
3	42	48	N	10.35	-	Yes	14.4	-	-	247
4	8	20	N	5.3	-	Yes	6.1	288	<30	276
5	13	18	N	1	-	No	12.5	-	-	311
6	Above 60	Above 60	N	12.37	-	No	10.1	-	-	625
7	Above 60	Above 60	N	1.66	1.043	Yes	15.7	-	-	199

Normal range, Urine Protein <150 mg/day; Hemoglobin, both sexes 12.0-17.5 g/dL; LDH 135 to 225 U/L; Haptoglobin 30 to 200 mg/dl; Platelets 150 to 400 platelets × 10^9^/L.

Abbreviations: eGFR, estimated glomerular filtration rate; LDH, lactate dehydrogenase; SCr, serum creatinine; PLT, platelets.

### Clinical Summaries

#### Patient 1

A 37 year old male with history of HTN and HLD presented to the UCI medical center (UCIMC) for headache, blurry vision, and elevated systolic blood pressure (SBP 200). He was found to have nephrotic range proteinuria and was admitted for hypertensive emergency. Renal biopsy confirmed IgA nephropathy with extensive fibrosis and concurrent TMA. The patient was started on dialysis, along with carvedilol 25 mg BID, hydralazine 100 mg TID, and nifedipine 90 mg QD for blood pressure control to be continued. Two years later, the patient successfully received a renal transplant. At 6 month follow-up, the patient’s allograft is functioning well with an immunosuppressive regimen of tacrolimus 5 mg QD, myfortic 360 mg BID, and prednisone 5 mg QD. Post-transplant, his nifedipine was continued at a lower dose of 30 mg QD for hypertension but his carvedilol and hydralazine were stopped. No family history of renal conditions. No tobacco, alcohol, or drug use.

#### Patient 2

A 37 year old female presented to UCIMC from her optometry clinic after being found to have bilateral retinal hemorrhages and disk swelling following 2 weeks of blurry vision in May 2020. At the ED, she was found to be severely hypertensive (BP 245/165) and was admitted for hypertensive emergency. During admission, renal biopsy confirmed IgA nephropathy and chronic active TMA. She was started on dialysis the same month along with carvedilol 25 mg BID. In 2023 the patient received a renal transplant, with the current immunosuppressive regimen consisting of prednisone 2.5 mg QD and tacrolimus 5 mg QD. Family history was positive for diabetic nephropathy but no other renal conditions. The patient endorsed occasional alcohol use; denied smoking or drug use.

#### Patient 3

A 29 year old male with a history of HTN presented to UCIMC from ophthalmology clinic for blurry vision and hypertension (systolic BP >180). He was admitted for hypertensive emergency and suspected glomerulonephritis February 2024. Inpatient biopsy was planned but as blood pressure was not maintained below 140/90 despite nifedipine, carvedilol, and hydralazine, he was discharged with outpatient follow-up. One month later, renal biopsy confirmed IgA nephropathy with TMA. Upon his diagnosis, he started hydralazine 50 mg BID, nifedipine 60 mg BID, and prednisone 40 mg BID. In 2025 the patient continued follow-up with an outside nephrologist and unfortunately further records are unavailable. No family history of renal disease. Endorsed occasional alcohol use, denied smoking or drug use.

#### Patient 4

A 47 year old patient with a history of newly diagnosed hypertension on amlodipine presented to UCIMC with heavy menses, fatigue, and dyspnea. She was found to have severe anemia (hemoglobin 6 g/dL) and nephrotic-range proteinuria requiring admission in June 2023. Renal biopsy confirmed IgA nephropathy with TMA, and the patient subsequently started dialysis the same month. Nephrology started the patient on furosemide, sodium bicarbonate, and vitamin D, along with hydralazine 25 mg Q8 hours PRN and metoprolol 25 mg BID for blood pressure control. The patient was maintained on a lisinopril 20 mg QD routine from May to June 2024 where it was discontinued following admission for aortic intramural hematoma. Metoprolol was discontinued August 2025 status post aortic dissection the same month; the patient was switched to carvedilol. Family history was significant for a brother 12 years younger with ESRD on HD (unknown etiology, renal biopsy inconclusive). No tobacco, alcohol, or drug use.

#### Patient 5

A 42 year old male with a history of HTN and self-reported history of autoimmune kidney disease (unknown type) who was visiting from out of town presented to UCIMC with chest pain and shortness of breath February 2023. He was found to be hypertensive (systolic BP in the 190 seconds) with new onset heart failure and AKI. While in admission, renal biopsy confirmed IgA nephropathy and acute-on-chronic TMA. The patient was started on hydralazine 25 mg Q8 Hr, carvedilol 25 mg BID, and nifedipine 30 mg QD for blood pressure control. The patient returned to his home city following discharge, where he established nephrology follow-up. Family history was pertinent for a sister in her 30s with an autoimmune condition (unknown type) but negative for other family renal history. No tobacco, alcohol, or drug use.

#### Patient 6

A 54 year old male with history of T2DM and HTN presented to UCIMC with worsening shortness of breath and extremity swelling. He was admitted for fluid overload and anasarca with course complicated by acute hypoxic respiratory failure due to acute pulmonary edema. He underwent diuresis with improvement. Renal biopsy during admission found focal segmental glomerular sclerosis, IgA nephropathy, and chronic TMA. He was started on nifedipine 30 mg QD, metformin 500 mg BID, aspirin 81 mg QD, and bumetanide 1 mg QD with adequate blood pressure control. Unfortunately, shortly afterward the patient was lost to follow up so further information on his case is unavailable. Family history was negative for renal conditions. No tobacco, alcohol, or drug use.

#### Patient 7

A 45 year old male with a history of vitamin D deficiency and HLD presented to the UCI nephrology clinic upon referral from primary care for proteinuria and microscopic hematuria in 2017. The patient endorsed mild rare orthostatic dizziness but was otherwise asymptomatic. Urology diagnosed the patient with nutcracker syndrome, which was initially considered to be the cause of his hematuria and proteinuria given his normal renal function and blood pressure. However, due to worsening proteinuria, renal biopsy was performed 2 years later which confirmed diagnosis of IgA nephropathy with TMA. The patient was treated with a course of high-dose steroids with stabilization of his proteinuria. ACEi/ARBs were attempted for nephroprotection, but the patient was unable to tolerate them due to a side effect of dry cough. Two years later, in March 2021, the patient presented with an IgA nephropathy flare (proteinuria 2.4 g/24 hours), prompting initiation of plaquenil 200 mg QD (unable to tolerate higher dose due to gastrointestinal side effects) and benazepril 2.5 mg QD. Seven months later, he stopped the plaquenil and benazepril, as blood pressure trended lower. The patient then started farxiga 10 mg QD based on positive findings in the literature. In December 2022, the patient restarted benazepril 2.5 mg QD and was able to tolerate it well; in June 2024, this was increased to 5 mg QD. In January 2025, the patient continued benazepril 5 mg and farxiga 10 mg QD. No family history of renal conditions. Endorsed alcohol use (1-5 drinks/week); denied tobacco or drug use.

### Histopathological Findings and Complement Assay

Of the 7, 3 had ESRD with 2 having undergone successful RRT with 1 on hemodialysis as of January 2026. 2/7 of patients had low C3 levels (Table 3). Doubling of serum creatinine occurred in 1 patient at follow-up, coinciding with a T2 finding.

**Table 3.** Complement and Immune Protein Assay.

Patient #	C3 mg/dL	C4 mg/dL	CH50 %	Protein amount mg/dL
1	126	41	83.9	-
2	72	29	-	IgM: 700
3	122	27	77.8	-
4	78	22	-	-
5	125	33	>95	-
6	91	28	-	IgA:500 IgM:1020
7	81.6	19	-	-

Normal range, C3- 80 to 160 mg/dl; C4 10-40 mg/dL; CH50 30% to 50%; IgA 80 to 300 mg/dl; IgM 40 to 250 mg/dl.

Abbreviations: IgA, immunoglobulin A; IgM, immunoglobulin M.

Laboratory testing and histology showed that of those 6/7 of patients presented who were diagnosed with IgA and showing tubular atrophy/interstitial fibrosis (*T* >0), 5/6 of them were classified MEST-C T2. This is a finding consistent with >50% of renal cortex containing tubular atrophy or interstitial fibrosis.^5^ In addition, 5/7 of patients showed mesangial hypercellularity, 3/7 showed endocapillary hypercellularity, all showed segmental glomerulosclerosis, and 4/7 showed crescents. Mesangial hypercellularity with cellular crescents was found in 4/7, with segmental fibrous crescent in 1/7, and with segmental glomerulosclerosis in 2/7 patients with mesangial IgA deposits.

**Figure 1.**
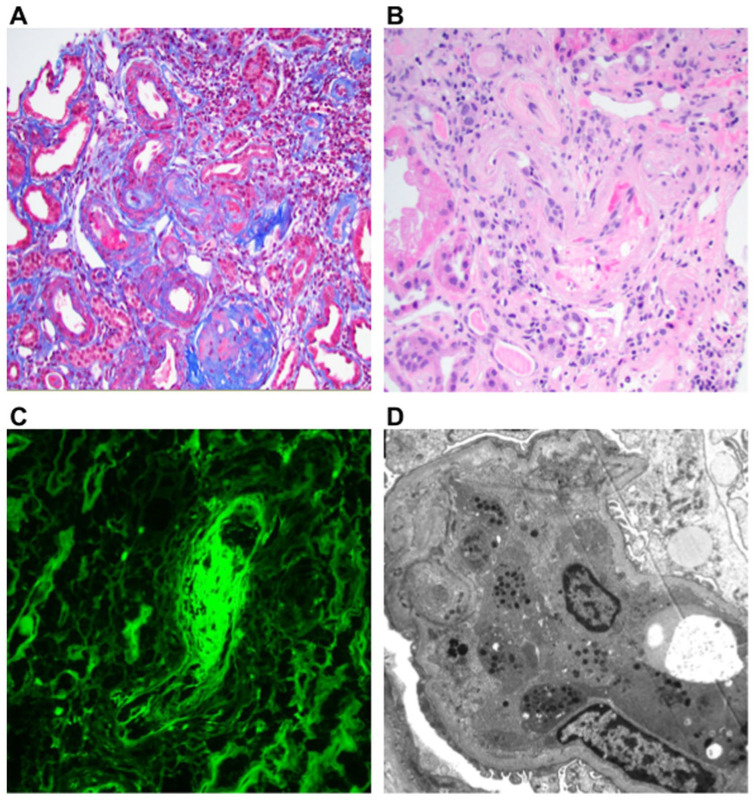
(A) Patient 1 acute thrombosis of an arteriole trichrome stain x200, (B) patient 5 acute thrombosis of an arteriole hematoxylin & eosin stain x400, (C) patient 6 luminal fibrinogen immunofluorescence staining of an arterial thrombus x400, and (D) patient 7 platelet thrombus in glomerular capillary lumen electron micrograph x1100. Figures are grouped by common findings to demonstrate trends.

As for lesions associated with TMA (Figure 1): acute arteriolar thrombosis was detected in 5/7 cases, while glomerular platelet thrombosis was identified in 1/7. The prevalence of TMA features in our cohort (5/7 cases, 71% with arteriolar thrombosis) is higher than most reported IgAN TMA series, which range from 13% to 53% depending on patient selection and pathology definition, though comparable to the 71% with uncontrolled hypertension reported by El Karoui et al , suggesting our cohort represents a more severe IgAN TMA phenotype.^6-11^

All cases were categorized for Oxford MEST-C classification, and histological features were noted (Table 4). Most patients had a high degree of chronic changes such as global glomerulosclerosis and interstitial fibrosis/ tubular atrophy. Additionally, moderate to severe arteriosclerosis and arteriolar hyalinosis were common features.

**Table 4.** Histology Summary.

Patient Number	M	E	S	T	C	TMA features	Other pathological findings	Glomerular immunofluorescence staining intensity
1	0	0	1	2	0	Focal arteriolar thrombosis	80% IFTA 88% GSG ARS - none AH - severe	IgA (4+), IgM (2+), C3 (2+), kappa light chain (2+), lambda light chain (2+)
2	1	1	1	2	1	Rare arteriolar thrombus, mucoid intimal edema and endotheliosis of arteries and arterioles, onion skin change in arterioles	70% IFTA 72% GSG ARS - severe AH - severe	IgG (trace), IgA (3-4+,), IgM (trace), C3 (3-4+), kappa light chain (2-3+), lambda light chain (3-4+)
3	1	1	2	1	1	Rare arteriolar thrombus. Onion skin changes in arterioles. Rare glomerular fibrin tactoid in EM studies	40% IFTA 39% GSG ARS - moderate AH - severe	IgG (trace), IgA (4+), IgM (1+), C3 (4+), kappa light chain (4+), lambda light chain (4+)
4	1	0	1	2	1	Focal arteriolar thrombosis. Mucoid intimal edema in arterioles. Onion skin change in arterioles	60% IFTA 73% GSG ARS - severe AH - severe	IgA (4+), IgM (1+), C3 (1-2+), kappa light chain (2+), lambda light chain (3+).
5	1	0	1	2	0	Focal arteriolar thrombosis	>50% IFTA 30% GSG ARS - moderate AH - moderate	IgA (4+), IgM (1-2+), C3 (4+), kappa light chain (1+), lambda light chain (4+).
6	0	0	1	2	0	Single arteriolar thrombus. Rare glomerular capillary wall double contours	60 IFTA 41% GSG ARS - moderate AH - severe	IgG (1-2+), IgA (2-3+), kappa light chain (1-2+), lambda light chain (2+), and C3 (2-3+)
7	1	1	1	0	1	Focal glomerular capillary loop thrombosis	<5% IFTA 8% GSG ARS - none AH - none	IgA (4+), IgM (1+), C3 (3-4+), kappa light chain (2-3+), lambda light chain (4+)

Oxford Elements are as follows: (M) mesangial hypercellularity; (S) segmental glomerulosclerosis; (E) endocapillary hypercellularity; (T) and (IFTA) tubular atrophy/ interstitial fibrosis; (C) crescents. IgA intensity on immunofluorescence is scaled as trace, +1 (mild), +2 (moderate), +3 (intense), +4 (glossy/radiant). Globally sclerotic glomeruli (GSG); % of total glomeruli completely scarred. Arteriosclerosis (ARS); none, moderate, and severe. Arterial hyalinosis (AH); none, moderate, and severe. These data were interpreted by a fellowship-trained renal pathologist.

Most biopsies showed at least 2 + immunofluorescence intensity on C3 stain. Patients *2, 4*, and *7* had low serum levels C3. And patient *1* showed high levels of C4d and elevated CH50. In patients with high C4d *or* low C3 *or* abnormally high CH50, 2 were on hemodialysis and 1 was on ACEi, 3 of the 4 were Oxford T2: >50% of renal cortex containing tubular atrophy or interstitial fibrosis. In patients with normal complement levels but abnormal IgM and IgA (patient *6*), T2 was also observed. Patient *3* showed elevated CH50.

## Discussion

IgA nephropathy is the most common primary glomerulonephritis.^12^ Disease-causing candidates are thought to include the complement factor H gene on chromosome 1q32 among others, with development associated with Gal-deficient *O*-glycans of IgA1 causing accumulating and leading to immune response and renal injury.^12^

The cause of IgAN, albeit multifactorial, is documented though often deemed idiopathic in the clinical setting. In one report, progressive IgAN and renal deterioration uncontrolled on immunosuppressive therapy was treated with the C5-inhibitor eculizumab to improve SCr and decrease proteinuria.^13^ Multiple systematic reviews^14-16^ describe the role of complement pathology in IgAN progression, while differentiating that advanced complement testing beyond just C3 and C4d levels are needed to determine this role; while also noting the importance of complement staining in IgAN renal biopsies.^14^ Elevated IgM mesangial deposition has been noted as an independent risk factor to complement dysregulation^14^ of poor renal outcomes and is observed in various systemic diseases.^17^ In a systematic review of Oxford MEST-C,^18^ higher T score was found most often related to worse clinical outcomes of all MEST-C elements, though not without imprecision.

### Complement Activation Links IgAN and TMA?

The association between complement activation and the IgAN TMA phenotype in our cohort is supported by both serologic and tissue findings. Tissue complement assessment via immunofluorescence showed that all biopsies exhibited at least 2+ C3 staining intensity on mesangial deposits, consistent with complement deposition at the site of injury.^6,19,20^ This is significant, as mesangial C3 deposition ⩾2+ has been independently associated with worse renal outcomes and more severe pathologic lesions in IgAN, and specifically with the development of microangiopathy in IgAN when combined with C4d deposition.^8,11,19-21^ In contrast, serologic complement markers provided a more variable picture: 2/7 patients demonstrated low serum C3 levels, 1/7 showed elevated C4, and 1/7 had abnormally high CH50, while 2/7 exhibited elevated serum IgM and/or IgA (Table 3). While serum complement abnormalities have prognostic value in IgAN, they are less sensitive than tissue markers for detecting local complement activation.^15,20-22^ The conflict between universal tissue complement deposition and selective serum abnormalities in our series underscores that complement activation in IgAN TMA is predominantly a local, tissue phenomenon rather than systemic.^20^ This distinction is critical, as markers of complement activation in kidney biopsy specimens including C3, C4d, and terminal complement components, have been shown to associate with disease activity and predict poor outcomes in both IgAN and specifically in complement-mediated microangiopathy within IgAN.^6,8,11,19,22^ The consistent tissue complement deposition across our cohort, coupled with the high prevalence of chronic changes (T2 lesions in 5/6 patients), suggests that ongoing alternative and lectin pathway activation may contribute to both the TMA phenotype and fibrosis observed in these patients.^20,23,24^

### Limitations

The major limitation is small sample size. Comprehensive complement system investigation with genetic testing is also lacking in our study. Instead, we aim to highlight the key features present in these case series that may be indicative of a larger trend and warrant further study.

## Conclusion

Complement therapy for IgAN TMA remains largely unexplored, with current evidence limited to case reports and extrapolation from broader IgAN and secondary TMA literature. No randomized trials have specifically evaluated complement inhibition for the IgAN TMA phenotype. The KDIGO IgAN guidelines identify complement inhibition mechanisms and optimal treatment combinations as prioritized research areas but provide no specific recommendations for the TMA subset.^25^ The strongest evidence comes from complement mediated TMA in other settings.

The KDIGO lupus nephritis guidelines^26^ recommend eculizumab (a C5 inhibitor) for complement mediated TMA that is refractory to plasma exchange and immunosuppression, noting a 68% resolution rate in secondary aHUS and complete kidney recovery in 4 of 5 plasma-resistant patients treated with eculizumab.

While these data derive from TMA with lupus rather than IgAN TMA, they establish proof of concept that complement blockade is efficacious in secondary microangiopathic processes.

For IgAN specifically, recent advances in complement inhibition show promise but lack TMA data. Iptacopan, an oral factor B inhibitor targeting the alternative pathway, recently received FDA approval for IgAN based on a 38.3% proteinuria reduction and demonstrated alternative pathway blockade via decreased urinary membrane attack complex.^27,28^ The trial showed consistent efficacy across inflammatory phenotypes, including Asian patients observed to have more severe disease.^28,29^ However, patients with concurrent TMA were not separately analyzed, and the trial excluded those with eGFR <30 ml/min/1.73 m^2^: the population inundated with IgAN TMA patients.^30^ Case reports describe successful eculizumab use in progressive IgAN unresponsive to immunosuppression, with improvements in creatinine and proteinuria.^31,32^ Given the tissue complement activation (mesangial C3 ⩾2+) in our IgAN TMA cohort and associations with microangiopathy with C4d deposition, and that alternative pathway dysregulation drives both IgAN progression and complement mediated TMA, targeted complement inhibition tracks mechanistically.^23,31-33^

Some practical considerations might include the lack of diagnostic biomarkers distinguishing complement mediation from other TMA subtypes in IgAN, the need for meningococcal vaccination with C5 inhibitors, and uncertainty regarding optimal prescription (eg, proximal pathway inhibition with iptacopan vs terminal pathway blockade with eculizumab).^27,33^

The presence of TMA in IgAN raises important diagnostic and prognostic questions that justify ongoing discussion, particularly of complement dysregulation.
